# Interlayer Strength of 3D-Printed Mortar Reinforced by Postinstalled Reinforcement

**DOI:** 10.3390/ma14216630

**Published:** 2021-11-03

**Authors:** Jihun Park, Quang-The Bui, Jungwoo Lee, Changbin Joh, In-Hwan Yang

**Affiliations:** 1 Department of Civil Engineering, Kunsan National University, Kunsan 54150, Korea; jhpark3@kunsan.ac.kr (J.P.); tqbui93@gmail.com (Q.-T.B.); 2 Department of Infrastructure Safety Research, Korea Institute of Civil Engineering and Building Technology, Goyang 10223, Korea; duckhawk@kict.re.kr (J.L.); cjoh@kict.re.kr (C.J.)

**Keywords:** 3D-printed mortar, reinforcement, interlayer, bonding strength, curing conditions

## Abstract

This work was designed to evaluate the interlayer strength of 3D-printed mortar with postinstalled interlayer reinforcement. Two methods of postinstalled interlayer reinforcement were considered according to the amount of overlapping. The first method did not include overlapping of the interlayer reinforcement, while the second method included overlap lengths of 20 and 40 mm. Additionally, two different curing conditions were considered: air-curing conditions and water-curing conditions. The compressive, splitting tensile, and flexural tensile strengths of 3D-printed mortar specimens with different reinforcement methods and curing conditions were investigated under three loading directions. The three loading directions were defined based on the three planes of the printed specimens. The compressive, splitting tensile, and flexural tensile strengths were dependent on the loading directions. In particular, the splitting and flexural tensile strengths decreased considerably when tensile stresses acted on the interlayers of the 3D-printed mortar specimens. However, when longitudinal interlayer reinforcement penetrated the printed layers, the flexural tensile strength or interlayer bonding strength of the printed specimens increased significantly at the interlayers. In addition, mortar specimens reinforced with overlap lengths of 20 and 40 mm were investigated in this study. The flexural tensile strength or interlayer bonding strength of 3D-printed mortar decreased after treatment under air-curing conditions because the interlayers of the printed mortar formed more pores under these conditions and were more vulnerable under loading. Finally, the findings of this study suggested that interlayer reinforcement is a potential method for improving the interlayer bonding strength of 3D-printed mortar.

## 1. Introduction

Recently, additive manufacturing techniques have received more attention in the construction industry [1,2,3]. These techniques involve the 3D printing of objects through the deposition of mortar filaments. Anisotropic material properties of 3D-printed mortar resulting from the deposition of layers should be considered in the design of 3D mortar printing. In addition, loading directions have a significant influence on the material properties of 3D-printed mortar [4,5,6,7,8]. In particular, the bonding and flexural tensile strengths of interlayers in 3D-printed mortar decrease under load application parallel to the interlayer interface. The interlayer bonding strength plays an important role in determining the structural behavior of the 3D-printed structure.

Several studies designed to improve the interlayer bonding strength of 3D-printed mortar have been conducted [9,10,11,12]. The addition of fiber to 3D-printed mortar enhanced the interlayer bonding strength of the mortar [13,14]. Shakor et al. [13] found that the strength properties of 3D-printed mortar were improved by the presence of chopped glass fibers. Ding et al. [14] analyzed the influence of polyethylene (PE) fiber lengths and contents on the mechanical properties. Their study showed that the addition of PE fibers to 3D-printed mortar improved the flexural strength and postpeak behavior of the printed specimen. Additionally, Hambach and Volkmer [15] investigated the effect of fiber orientation control during the printing process on the strength properties of 3D-printed mortar. According to their study, alignment of the fibers during the 3D-printing process increased the strength of 3D-printed specimens.

Moreover, two typical methods are used to install reinforcement into a 3D-printed structure: the preinstalled and postinstalled reinforcement methods [16]. In the preinstalled reinforcement method, reinforcements are fabricated and installed in place before the printing process is finished. This method is favorable for printing vertical elements such as walls and columns, but it poses difficulties in building nonvertical elements. Movement of the printing nozzle is difficult due to the interference between preinstalled reinforcements and the nozzle, and therefore, a special nozzle design is required. The shape of the printing nozzle also affects the width of the printed filaments [17].

In the postinstalled reinforcement method, steel reinforcing bars are installed into mortar layers after the printing process is finished. This method is more common than preinstalled reinforcement. Accordingly, the effect of this method on the bonding strength has been investigated by several researchers. Hass and Bos [18] introduced a new reinforcement method using screw-type reinforcements for 3D-printed mortar. In this study, the failure patterns of three-point bending and pull-out tests implied that screw-type reinforcements improved the interlayer bonding strength of the printed mortar. Another type of reinforcement was suggested in the study of Ma et al. [19]. A microcable was used to reinforce the 3D-printed mortar. The test results showed that the flexural strength and postcracking softening behavior were improved in printed mortar reinforced by microcables. Bester et al. [20] evaluated the postcracking behavior of printed mortar reinforced by straight steel fibers. They reported that the addition of straight steel fibers into mortar filaments enhanced the ductile postcracking behavior of printed mortar. Perrot et al. [21] also investigated the effect of nail reinforcements on mortar strength. They concluded that the flexural tensile strength and ductility of the printed specimen were enhanced by adding nails into mortar layers. In addition, Wang et al. [22] suggested that alkali-resistant glass textile reinforcements showed potential for use in thin-shelled structural elements. The flexural strength, flexural stiffness, and postcracking ductility of casted and printed specimens were not significantly different in bending tests of the thin-casted and printed prismatic specimens used in their study. Most studies imply that reinforcements penetrating through interlayers provide a potential method to enhance the strength of 3D-printed mortar, and thus, the installation of interlayer reinforcements may be required. Bos et al. [23] concluded that the addition of cable reinforcements significantly improved the precracking and postcracking strength of 3D-printed specimens.

Additionally, the interlayer bonding strength of 3D-printed mortar can be influenced by curing conditions. The effects of curing conditions on the interlayer bonding strength have been reported in some studies [24,25]. Rashid et al. [24] investigated the effects of different curing conditions on the bonding strength of the interface between mortar and polymer cement mortar. An insignificant effect of moisture on the interlayer bonding strength was reported in the study. Meanwhile, Weng et al. [25] found that the interlayer bonding strength was improved significantly by water-curing and climate chamber-curing conditions. Therefore, previous results show that there is controversy regarding the effects of curing conditions on the bonding strength of the interface.

Although reinforcement methods that involve adding flexible fibers to mortar filaments to improve the bonding strength of 3D-printed mortar have been suggested, the addition of fibers may reduce the extrudability of printing filaments. Therefore, as an alternative, the postinstalled steel reinforcement method for interlayers is considered. Moreover, there is controversy regarding the effects of curing conditions on the interlayer bonding strength of 3D-printed mortar.

Therefore, this study was designed to investigate the tensile and bonding strength characteristics of 3D-printed mortar with postinstalled steel reinforcement at the interlayers. In addition, the effects of curing conditions on the strength of the 3D-printed mortar with postinstalled reinforcement were analyzed. Four 3D mortar structures were printed, and then, mortar specimens were extracted from the structures. Finally, the effects of the loading direction, overlap length of interlayer reinforcements, and curing conditions on strength properties were analyzed and compared by extensive testing.

## 2. Material and Mixing Proportions

The extrudability of 3D-printed mortar describes its ability to be continuously forced through the nozzle. Buildability refers to the resistance of deposited fresh mortar to deform during construction and the ability of the mortar to retain its extruded shape [26]. Extrudability and buildability are critical requirements for 3D-printed mortar in the fresh state. To achieve these requirements, the consistency and constituents of 3D-printed mortar mixtures should be considered. In this study, sand with particle sizes in the range of 0.16 to 0.2 mm was used. The binder adopted in this study was a combination of ordinary Portland cement (OPC), silica fume (SF), and class C fly ash (FA). The details of the mixing proportions are shown in Table 1. The OPC had a density of 3.14 g/cm^3^, and the FA had a density of 2.26 g/cm^3^. SF with a SiO_2_ content of 91.3% and a density of 2.81 g/cm^3^ was added to the mixture. A high-performance water-reducing agent (HWRA) was added to the mortar mixture to secure a target water–binder ratio of 0.25. The addition of an HWRA also improved the extrudability and strength of the 3D-printed mortar. Additionally, a viscosity agent was added to the mixture to improve the viscosity of the mixture and prevent segregation of the mixture components. The viscosity agent controlled the drying shrinkage of the mortar filament because it prevented water evaporation [27,28]. The use of an accelerator improves the green strength of 3D-printed mortar at an early curing age and, accordingly, its buildability. However, accelerator use may adversely affect the extrudability of 3D-printed mortar; therefore, an accelerator was not used in the mixture in this study.

The cementitious paste volume was calculated and is provided in Table 1. The cement or cementitious paste volume contributes to the properties of fresh and hardened cementitious materials [29]. The workability of mortar and concrete depends on the cementitious paste volume [29,30]. The initial and final setting times of the 3D-printed mortar were approximately 500 and 660 min, respectively.

**Figure 1:**
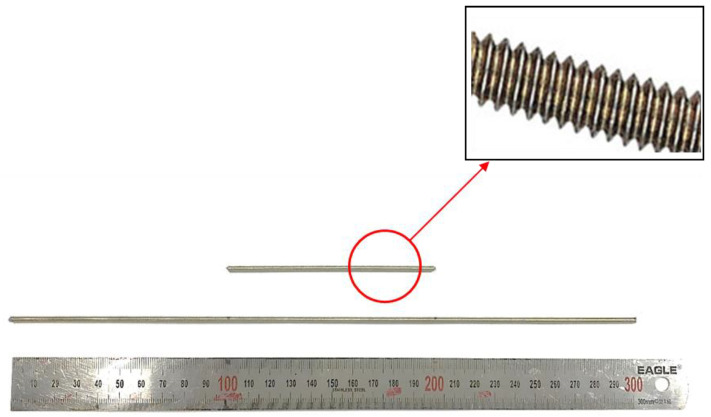
Interlayer reinforcements.

For prismatic specimens with dimensions of 50 × 50 × 300 mm^3^, as described in Section 3.2, interlayer reinforcement provides tensile reinforcement. For each prism section, the tensile reinforcement ratio was intended to be less than 1.0%. This implied that reinforcements with a diameter of less than 5 mm should be selected. Moreover, the use of reinforcements with a large diameter might induce excessive lateral deformation due to penetration of the reinforcements into the layers with a width of 50 mm. Therefore, a steel rod with a nominal diameter of 3 mm was used as the interlayer reinforcement element for 3D-printed mortar. Typical interlayer reinforcements with lengths of 100 and 300 mm are shown in Figure 1. The interlayer reinforcements had screws along the outer faces. The yielding and ultimate strengths of the interlayer reinforcements were 506 and 584 MPa, respectively.

## 3. 3D-Printing Program and Preparation of Specimens

### 3.1. 3D-Printing Method

**Figure 2:**
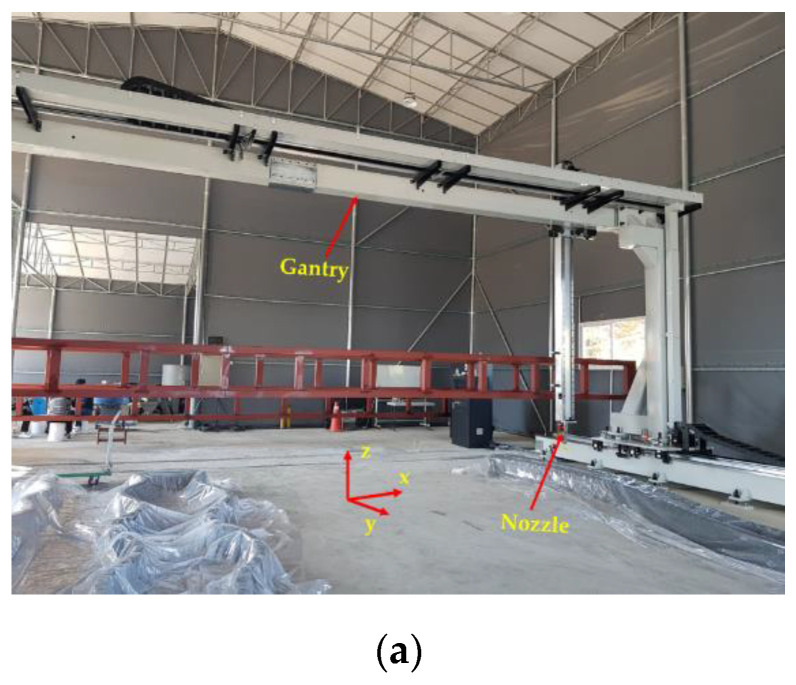

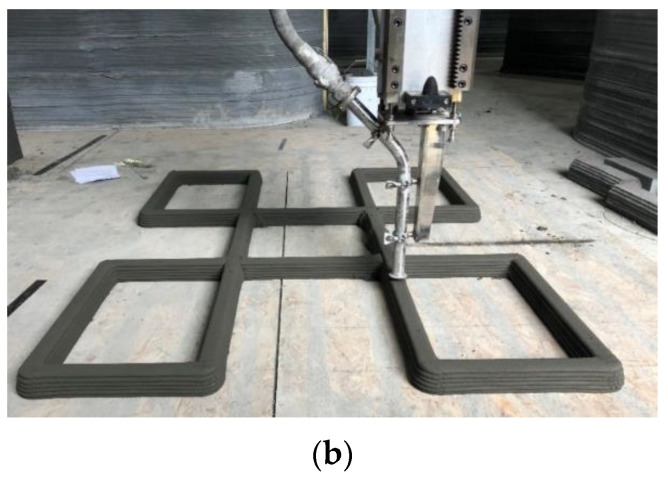
3D-printing system. (**a**) Printing gantry system; (**b**) Printing nozzle.

The printing gantry system (FP-7) used in this study, which moves in the X, Y, and Z directions, is shown in Figure 2. A mortar container was connected to a pump to convey the mortar filament into the printing nozzle (Güdel Lineartec Inc, Incheon, Korea), which was operated by a PC controller. A printing nozzle with a diameter of 40 mm was used to print the structures. The printing process included three stages: coordinate data preparation, mortar filament preparation, and mortar printing. In the coordinate data preparation stage, the printing path for each layer was generated by producing a G-code file for the printing process. In the mortar filament preparation stage, mortar was mixed and placed into the container. The fresh mortar was transferred smoothly through the pump–pipe–nozzle system to extrude the mortar filament from the container. The structures were printed in one batch. Therefore, there were no break times in printing or cold joints in the printed structures.

Finally, the operation of the printing process, which incorporated the nozzle height, printing speed, and printing directions, was controlled by the controller system. The nozzle elevation could be adjusted in the Z direction during the printing process.

**Figure 3:**
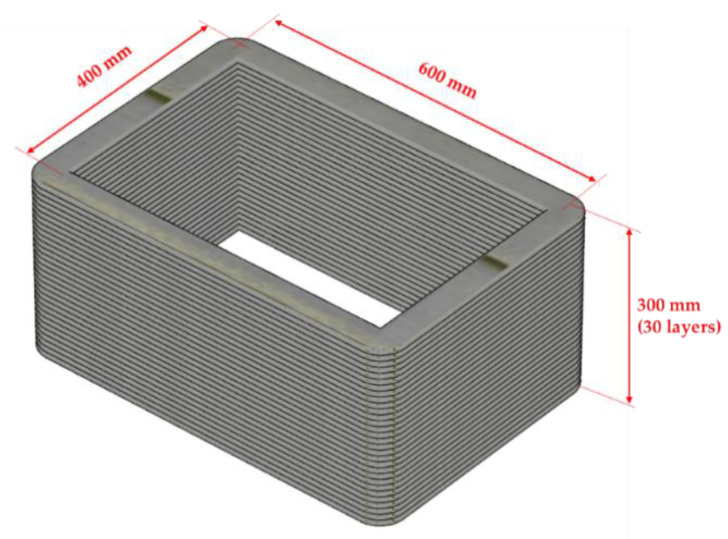
Illustration of a printed column.

To investigate the effects of reinforcements with different overlap lengths on the strength of 3D-printed mortar, four rectangular columns were printed. The four 3D-printed mortar structures were printed using the same printing process and had the same dimensions, as shown in Figure 3. The center-to-center distances between the two walls along the short and long directions were 400 and 600 mm, respectively. The four structures were laminated with up to 30 layers at a printing nozzle speed of 3 mm/s. The height of each layer was approximately 10 mm; thus, the total height of each column was considered to be 300 mm.

**Figure 4:**
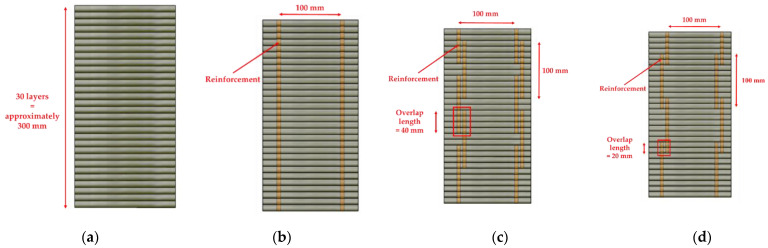
Schematics of interlayer reinforcements. (**a**) S1; (**b**) S2–300; (**c**) S3–40; (**d**) S4–20.

The schematics of the interlayer reinforcement types in the printed structures are shown in Figure 4. The four structures were distinguished according to the presence of interlayer reinforcements and the overlap length, and they were identified as S1, S2–300, S3–40, and S4–20. Structure S1 was a control structure and thus did not contain any rebar at the interlayers, as shown in Figure 4a. However, in structures S2–300, S3–40, and S4–20, screw-type rebar penetrated through the fresh mortar layers regularly along the long walls in each structure, as shown in Figure 4.

Structure S2–300 was printed with the same rectangular shape as structure S1, but the two long walls of structure S2–300 were reinforced by interlayer reinforcements with a length of 300 mm, as shown in Figure 4b. The reinforcements penetrated through layers at a spacing of 100 mm in the printing direction.

Structures S3–40 and S4–20 were also 30-layer printed column structures with the same dimensions as structure S1 and reinforced by interlayer reinforcements with overlap lengths of 40 and 20 mm, respectively. To date, there have been no recommendations for the overlap length of interlayer reinforcements because standards or guidelines for interlayer reinforcement methods in 3D-printed structures have not been provided. The thickness of a layer in the 3D-printed structures in this study was 10 mm; thus, the overlap length was considered in terms of a layer with a thickness of 10 mm. Therefore, overlap lengths of 20 and 40 mm were finally selected, which corresponded to two and four times the thickness of a layer, respectively, in this study. Interlayer reinforcements with a length of 100 mm were applied in structure S3–40 with an overlap length of 40 mm, as shown in Figure 4c.

The locations of reinforcements were determined along the centerline of the printed layer by using a scale and then manually marked on the layer. Therefore, reinforcements were located at a spacing of 100 mm as precisely as possible. Interlayer reinforcements were manually penetrated through the printed layers. The first interlayer reinforcements were installed through the mortar layers after ten mortar layers were printed.

The method applied for the first interlayer reinforcements was also used to determine the locations of the second interlayer reinforcements at a spacing of 100 mm. The second interlayer reinforcements were placed at a distance of 5 mm from the first interlayer reinforcements. The second interlayer reinforcements were installed through ten mortar layers after six additional mortar layers were printed. Finally, the reinforcements were spliced through four mortar layers to achieve the overlap length of 40 mm. This process was sequentially repeated until printing of the structure was complete.

**Figure 5:**
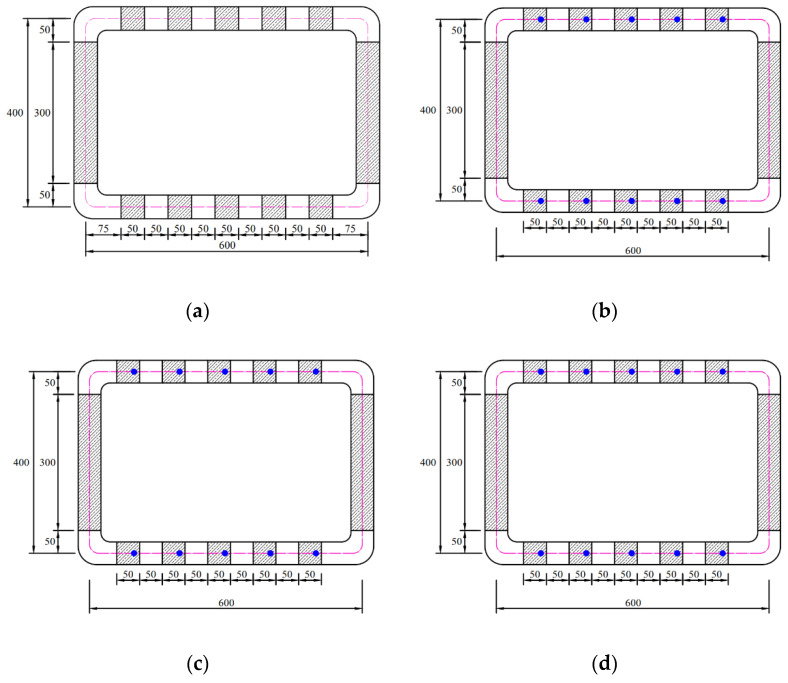
Details of the locations of reinforcements and extractions in mortar specimens. (**a**) S1; (**b**) S2–300; (**c**) S3–40; (**d**) S4–20.

**Figure 6:**
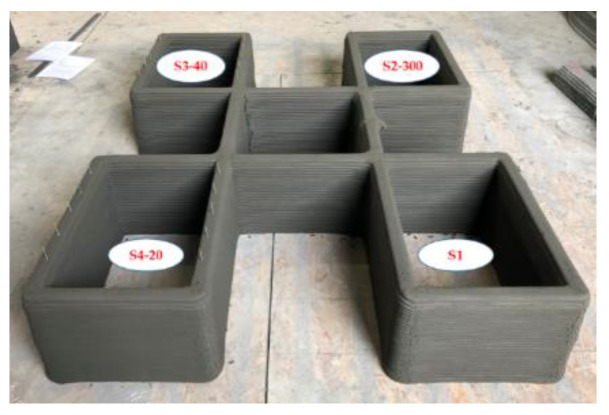
Printed structures.

**Figure 7:**
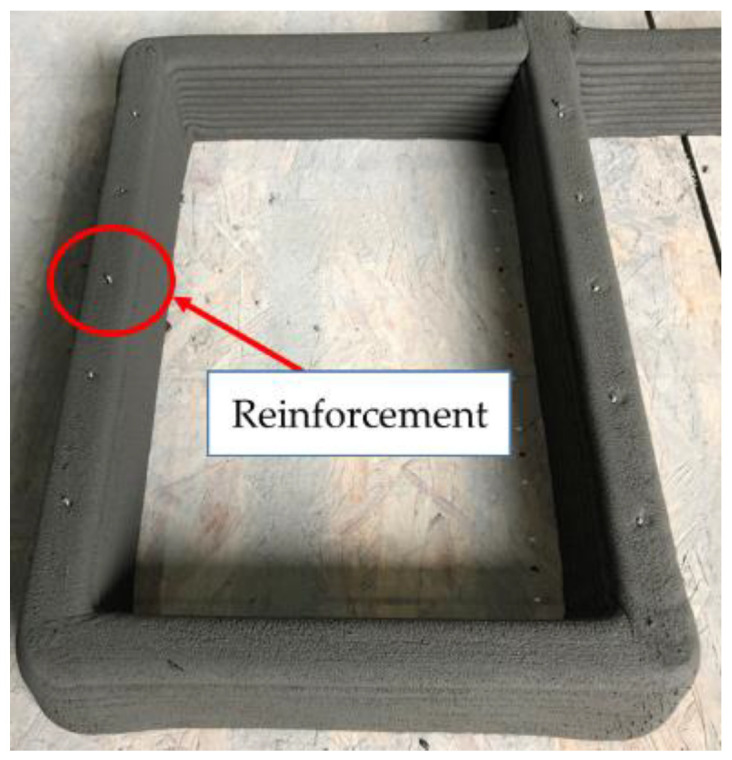
Penetration of reinforcements through layers.

Structure S4–20 also had the same dimensions as structure S1. Interlayer reinforcements with a length of 100 mm were applied in structure S4–20, with an overlap length of 20 mm, as shown in Figure 4d. The first interlayer reinforcements were installed through the mortar layers after ten mortar layers were printed. The second interlayer reinforcements were penetrated through ten mortar layers after eight additional mortar layers were printed. Thus, the reinforcements were spliced through two mortar layers to achieve the overlap length of 20 mm. This process was also sequentially repeated through 30 layers of printing. The details of the reinforcement locations along the walls in each structure are shown in Figure 5. In addition, the actual printed structures are shown in Figure 6, and Figure 7 presents the penetration of interlayer reinforcements through the printed layers.

### 3.2. Preparation of Specimens

3D-printed mortar can achieve high strengths to stabilize structures at early ages due to its short setting time. Several studies have reported that the green strength of 3D-printed mortar increased significantly after approximately 90 minutes of filament deposition [31,32,33]. Therefore, an extraction method used at early curing ages was adopted in this study. According to the test results of Chen et al. [34], the green strength of 3D-printed mortar increased by approximately 8 times at 4 h after printing compared to the strength at 1.5 h after printing. Additionally, several walls were printed preliminarily in this study. When the walls were cut at 4 h after printing, no significant deformation of the shape of the wall structures or segregation between interlayers was observed. Therefore, to prevent disturbing the interlayer bonding of the mortar at early curing ages, the walls of the printed structures were cut between five and six hours after finishing the printing process.

**Figure 8:**
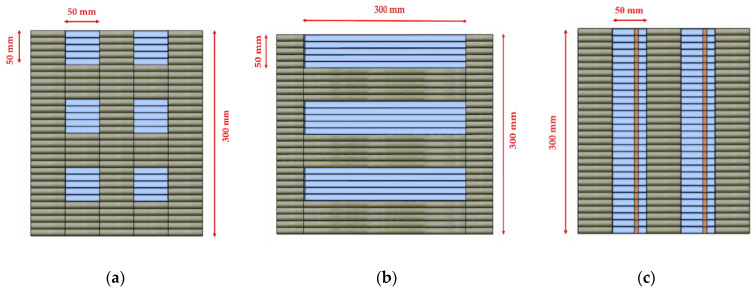
Extracting mortar specimens from the structures. (**a**) Cubic specimens for compressive and splitting tensile tests; (**b**) Prismatic specimens for flexural tensile tests under loading directions I and II; (**c**) Prismatic specimens for flexural tensile tests under loading direction III.

To obtain specimens for material tests, each wall of the printed structures was cut into cubic and prismatic specimens. A typical representation of the prismatic specimens extracted from the printed structures is shown in Figure 8.

For the compressive and splitting tensile tests, cubic specimens with dimensions of 50 × 50 × 50 mm^3^ were extracted from the printed walls of structure S1, as shown in Figure 8a. In addition, monolithic specimens with dimensions of 50 × 50 × 50 mm^3^ were fabricated for the compressive test. To obtain specimens for flexural tensile tests under loading directions I and II, prismatic specimens with dimensions of 50 × 50 × 300 mm^3^ were extracted parallel to the printing direction, as shown in Figure 8b. Cubic specimens with dimensions of 50 × 50 × 50 mm^3^ were used for the compressive and tensile splitting strength tests, and accordingly, prismatic specimens with dimensions of 50 × 50 × 300 mm^3^ were extracted to ensure equivalent dimensions of 50 × 50 mm^2^ between the cubic and prismatic specimens. For the flexural tensile test under loading direction III, prismatic specimens with dimensions of 50 × 50 × 300 mm^3^ were extracted perpendicular to the printing direction, as shown in Figure 8c. Interlayer reinforcements were located at the tensile zone in prismatic specimens extracted from the printed structures.

**Figure 9:**
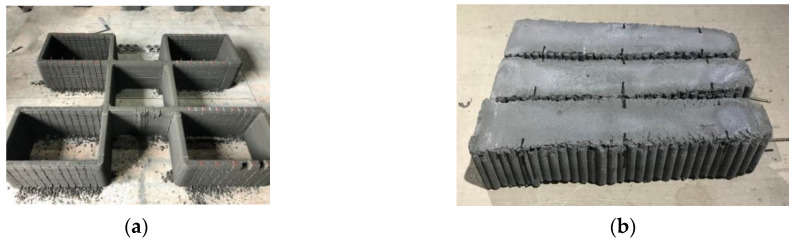
Process of extracting specimens. (**a**) Cutting process; (**b**) Cutting surface.

The actual process of extracting prismatic specimens from the structures is shown in Figure 9a. Each wall in the printed structures was cut with a steel cutter by hand. One day after cutting the printed structures, the specimens were extracted carefully by hand and cured under different curing conditions. The extracted specimens presented in Figure 9b show that cutting the structures did not induce shape deformation or segregation between interlayers. After the specimens were extracted from the 3D-printed structures, some specimens were cured in water at 24 ± 2 °C, and the other specimens were cured in air at room temperature for 28 days. After curing for 28 days, some prismatic specimens were cut into cubic specimens with dimensions of 50 × 50 × 50 mm^3^ for compressive and splitting tensile tests.

## 4. Method for the Evaluation of Mortar Strength

**Figure 10:**
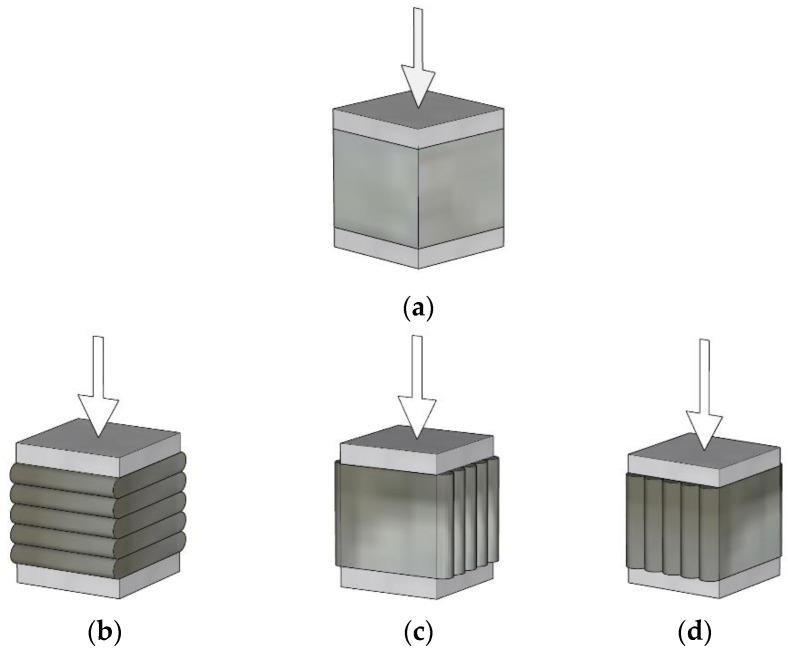
Compressive strength tests with different loading directions. (**a**) Monolithic specimen; (**b**) Loading direction I; (**c**) Loading direction II; (**d**) Loading direction III.

For the compressive strength test, monolithic and printed cubic specimens were tested. Three relative loading directions based on the planes of the printed specimens were considered, as shown in Figure 10, which are hereafter designated loading directions I, II, and III. The compressive strengths of the monolithic and printed specimens were calculated in accordance with ASTM C109/C109M-07 [35] as follows:

(1) $$f_{c}^{}\;=\;\frac{P}{b\times h}$$

where *b* is the width of the specimen section (mm), *h* is the height of the specimen section (mm), and *P* is the maximum load (N).

**Figure 11:**
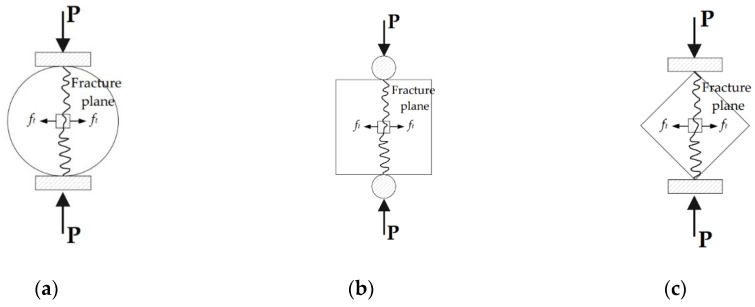
Test methods for splitting tensile strength. (**a**) Cylindrical specimen; (**b**) Cubic specimen (Case I); (**c**) Cubic specimen (Case II).

To estimate the splitting tensile strength of concrete, cylindrical or cubic specimens can be used. Cylindrical specimens are recommended in ASTM C 496/C 496M–04 [36], while cubic specimens or cylindrical specimens are recommended in BS EN 12390-6: 2009 [37] and ISO 1920-4: 2020 [38]. For these tests, two lines along which the load is to be applied are marked at the top and bottom of a specimen. The loading lines should be opposite from each other, and thus, connecting the extremities of the two loading lines defines the fracture plane of tensile splitting, as shown in Figure 11a for a cylindrical specimen and in Figure 11b for a cubic specimen. Additionally, a cubic specimen can be placed diagonally between two loading plates [39], and splitting tensile failure occurs along the diagonal plane between the two edges, as shown in Figure 11c.

**Figure 12:**
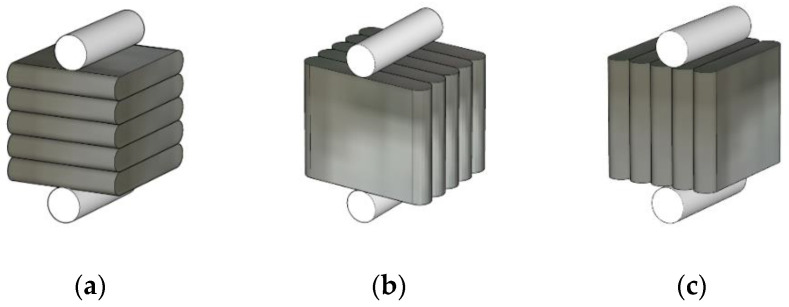
Splitting tensile strength tests with different loading directions. (**a**) Direction I; (**b**) Direction II; (**c**) Direction III.

Moreover, when mortar is cast monolithically in a mold, either of the two geometries for specimens can be selected. However, in obtaining specimens for the splitting tensile strength test, extracting cylindrical specimens from 3D-printed structures may cause difficulties in cutting the structures. Therefore, extracting cubic specimens from 3D-printed structures is preferred, and thus, cubic specimens were applied to estimate the splitting tensile strength of 3D-printed mortar under different loading directions in previous studies [4,6]. In this study, splitting tensile strength tests of cubic specimens were carried out under loading directions I, II, and III, as shown in Figure 12.

The splitting tensile strength test was performed by using the same uniaxial loading system applied in the compressive strength test. To induce cubic specimen splitting, specimens were loaded between steel rods in the splitting tensile strength test, while they were loaded between even steel plates in the compressive strength test. To avoid deformation of the steel rods under high-load conditions, the rods were made from hot-rolled steel. The applied load was controlled with a displacement rate of 0.8 mm/min. The splitting tensile strengths were calculated as follows:

(2) $$f_{t}\;=\;\frac{2\times P}{\pi \times l\times b}$$

where *l* is the line contact length of the load (mm), *b* is the width of the specimen section (mm), and *P* is the maximum load (N).

**Figure 13:**
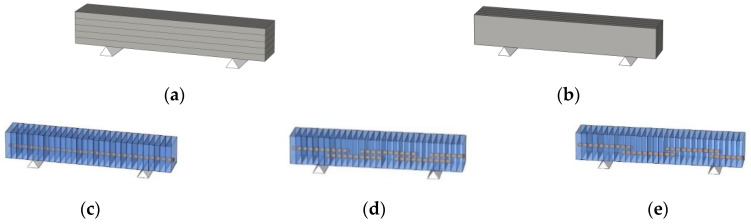
Flexural tensile strength tests under different loading directions. (**a**) Direction I (S1); (**b**) Direction II (S1); (**c**) Direction III (S2–300); (**d**) Direction III (S3–40); (**e**) Direction III (S4–20).

To measure the flexural tensile strength of the 3D-printed mortar, the control specimens were tested under loading directions I and II. Prior to the test in this study, separate 3D printing and strength test processes were performed preliminarily. The preliminary strength test showed that the flexural tensile strengths of specimens without reinforcement under loading direction III were less than 0.4 MPa; thus, these values could be disregarded. Therefore, a flexural tensile test under loading direction III was not performed for the unreinforced specimen in this study. The reinforced specimens were tested at the interlayers in direction III to evaluate the effects of the interlayer reinforcements on the interlayer bonding strengths, as shown in Figure 13.

A three-point bending test method to estimate the flexural tensile strength was selected in accordance with ASTM C348-18 [40]. In the three-point bending test, due to anisotropy resulting from the deposition of layers, the failure section of a prismatic specimen under loading direction III will be an interlayer section under a loading point. Meanwhile, the four-point bending test method can be applied to secure a constant-bending moment region between two loading points because the failure section of an isotropic prismatic specimen and the loading point section do not always coincide in the three-point bending test.

The flexural tensile strengths of the prismatic specimens were calculated as follows:

(3) $$f_{r}\;=\;\frac{3\times P\times l}{2\times b\times h^{2}}$$

where *l* is the distance between the supports (mm), *b* is the width of the squared section of the prismatic specimen (mm), *h* is the height of the squared section of the prismatic specimen (mm), and *P* is the maximum load (N).

The actual dimensions of each side of the test specimens should be considered because the cross-sectional areas depend on the bead width and layer height. Therefore, before performing the material tests, each side of the test specimens was measured in this study to obtain the actual cross-sectional area.

## 5. Test Results and Discussions

### 5.1. Compressive Strength

**Figure 14:**
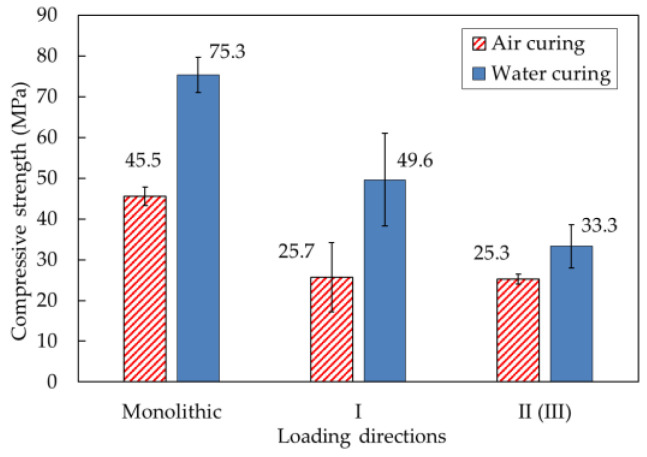
Comparison of the compressive strengths of mortar samples produced with different curing conditions.

The results of the mortar compressive strength tests under different loading directions are shown in Table 2 and Figure 14. As shown in Figure 10c,d, for the cubic specimen sections, the application of compressive loading in loading directions II and III was equivalent. Therefore, loading direction II could be considered the same as loading direction III, and compressive strength tests under loading directions II and III were not performed individually. Accordingly, the results of the compressive strength tests under loading direction II are also shown for loading direction III in the table.

**Figure 15:**
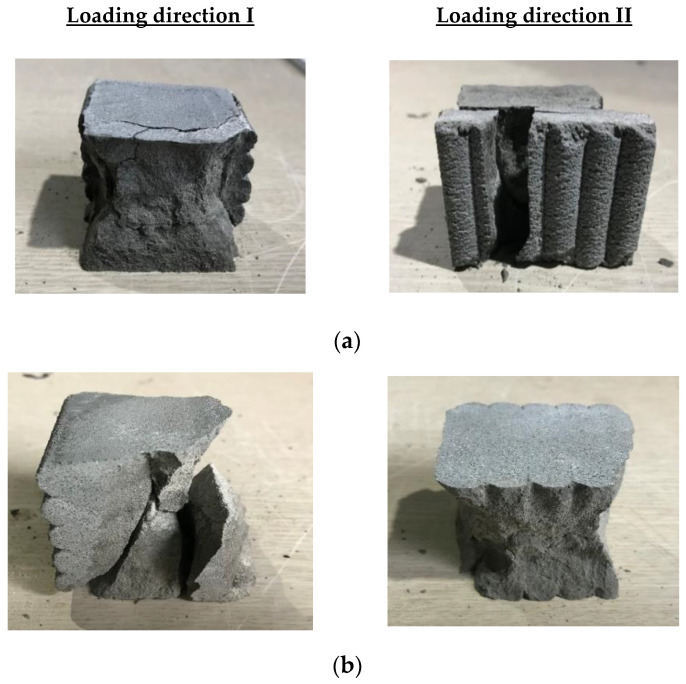
Compressive failure patterns of mortar samples under loading directions I and II. (**a**) Water-cured specimens; (**b**) Air-cured specimens.

For specimens produced with water curing conditions, the compressive strength of the monolithic specimen was 75.3 MPa and greater than that of the printed specimens. According to microscopic results, Nerella et al. [8] reported that weak interlayer bonding strength resulted from weak or cold joints at the interlayers. These weak interfaces induced long and wide separation between layers due to air enclosure at the interfaces. Therefore, the failure of printed mortar specimens might easily occur at the interlayer when a load is applied parallel or perpendicular to the interlayer joint. The test results in this study also indicated that the interlayer bonding strength affected the decrease in compressive strength of the printed specimens. Additionally, the mean compressive strengths of the water-cured specimens were 49.6 and 33.3 MPa in directions I and II (III), respectively. The compressive strengths of the water-cured specimens under loading direction II (III) were lower than those under loading direction I because the decrease in compressive strength under loading direction II depended on the interlayer bonding strength and the anisotropy of the printed specimens. The failure patterns of the water-cured specimens are presented in Figure 15a. Debonding of the interlayers under loading direction II eventually caused failure of the printed specimens.

For air-curing conditions, the compressive strengths of monolithic and printed specimens under different loading directions are also shown in Figure 14. For specimens produced with air-curing conditions, the monolithic specimen also exhibited approximately two times greater compressive strength than the printed specimens. The compressive strengths under loading directions I and II (III) of the printed specimens were 25.7 and 25.3 MPa, respectively. The difference between the compressive strengths of the printed specimens under the two different loading directions was not significant. Air-cured specimens that failed under loading directions I and II are shown in Figure 15b. In particular, failure due to debonding at interlayers under loading direction II was not observed with the naked eye, which indicated that the compressive strength of the printed specimen under loading direction II depended only slightly on interlayer bonding failure.

The differences in compressive strength between water-cured and air-cured specimens were significant. In particular, the compressive strengths of monolithic and printed specimens produced under water-curing conditions were approximately two times greater under loading direction I than those of monolithic and printed specimens produced under air-curing conditions. The study by Termkhajornkit et al. [41] reported that the hydration degree of fly ash-cement paste was improved under water-curing conditions and that the pozzolanic reaction of fly ash could occur with a small amount of water. Therefore, enhancement of the degree of hydration in the fly ash-cement paste under water-curing conditions enhanced the strength of the 3D-printed mortar.

### 5.2. Splitting Tensile Strength

**Figure 16:**
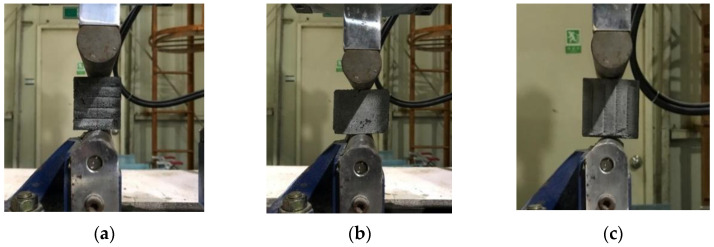
Splitting tensile strength tests under three loading directions. (**a**) Loading direction I; (**b**) Loading direction II; (**c**) Loading direction III.

**Figure 17:**
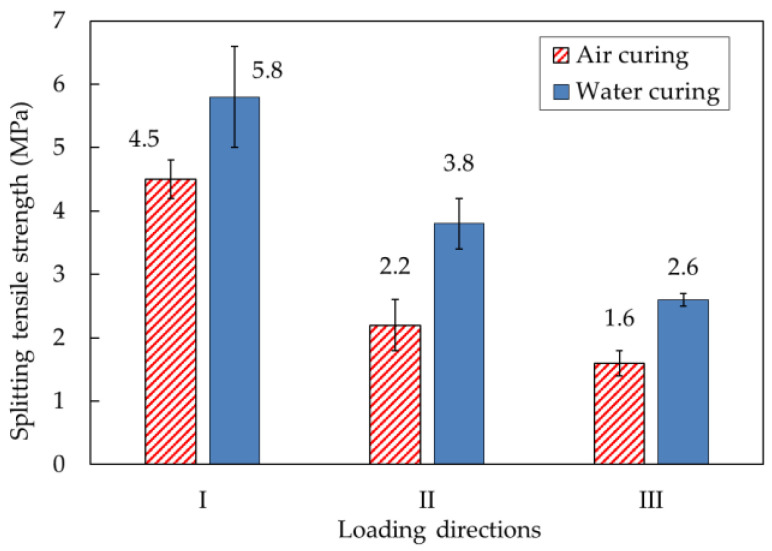
Comparison of the splitting tensile strengths of mortar samples produced under different curing conditions.

**Figure 18:**
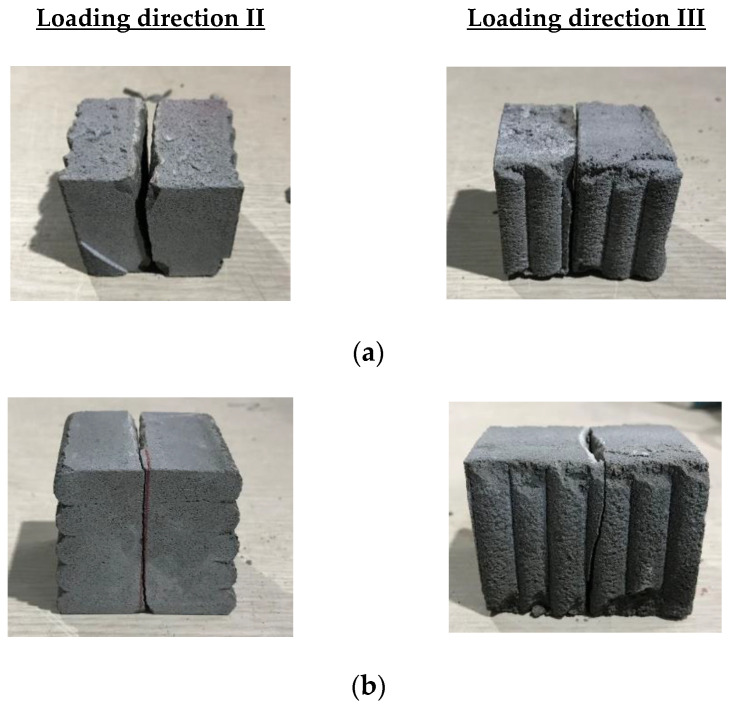
Splitting tensile failure patterns of mortar samples under loading directions II and III. (**a**) Water-cured specimens; (**b**) Air-cured specimens.

The actual splitting tensile strength tests under three loading directions are presented in Figure 16. Figure 17 shows the splitting tensile strengths of printed mortar specimens under the different loading directions. For specimens produced with water-curing conditions, the splitting tensile strengths under the three loading directions were 5.8, 3.8, and 2.6 MPa, respectively. These test results showed that the splitting tensile strength of the specimen was highly dependent on the loading direction. Moreover, the splitting tensile strengths of the specimens under loading direction III were lower than any other splitting tensile strength. The failure patterns of the water-cured specimens are shown in Figure 18a. The splitting tensile failure of the specimen under loading direction III occurred along the interlayer face, while the splitting tensile failure of the specimen under loading direction II occurred along the face perpendicular to the interlayer. Therefore, the splitting tensile strength under loading direction III was directly affected by the interlayer bonding strength of the specimen.

For specimens produced with air-curing conditions, the splitting tensile strengths of the air-cured printed specimens in the different directions are shown in Figure 17. The splitting tensile strengths of the air-cured specimens under the three loading directions were 4.5, 2.2, and 1.6 MPa, respectively. Just as the splitting tensile strength under water-curing conditions was affected by the loading direction, the splitting tensile strength under air-curing conditions was also affected by the loading direction. This phenomenon indicated that the splitting tensile strength of the 3D-printed specimens depended strongly on the anisotropy characteristics of the mortar. The failure of an air-cured specimen under loading direction III is shown in Figure 18b. Cracking was initiated at the interlayer, and finally, separation of the interlayer led to failure of the mortar. The failure observation indicated that the splitting tensile strengths of 3D-printed mortar specimens depended strongly on the anisotropy characteristics of the mortar and bonding properties at the interlayers.

Additionally, the splitting tensile strength after water curing was greater than that after air curing. Water curing of mortar promoted hydration of the mortar and thus improved the splitting tensile strengths of water-cured mortar specimens compared to those of air-cured mortar specimens. This implied that moist conditions would increase the splitting tensile strength of 3D-printed mortar structures at construction sites.

### 5.3. Flexural Tensile Strength

**Figure 19:**
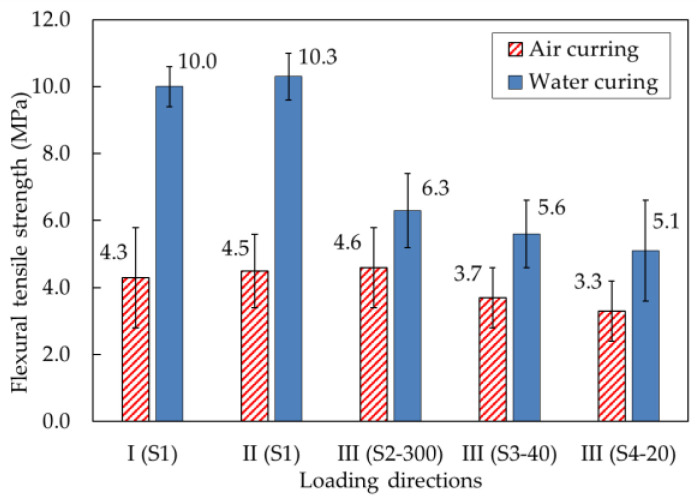
Comparison of the flexural tensile strengths of mortar samples produced under different curing conditions.

The flexural tensile strengths of the printed specimens under loading directions I, II, and III are shown in Figure 19. The specimens were reinforced in loading direction III by different reinforcement methods, which are identified as S2–300, S3–40, and S4–20 in Figure 4.

For specimens produced with water-curing conditions, the flexural tensile strength of 10.0 MPa under loading direction I was similar to the 10.3 MPa value under loading direction II. However, the flexural tensile strength under loading direction III was much lower than that under loading directions I and II. Under loading direction III, flexural tensile stresses occurred at the interlayers. The flexural tensile strength under loading direction III was closely related to the interlayer bonding strength, specifically at the interface between the printed layers. Accordingly, the printed interlayers could be separated in mortar failure under excessive tensile stresses.

With different interlayer reinforcements, the flexural tensile strengths of specimens S2–300, S3–40, and S4–20 were 6.3, 5.6, and 5.1 MPa, respectively, under loading direction III. The test results showed that the presence of reinforcements and different overlap lengths affected the flexural tensile strengths. Although the flexural tensile strengths of specimens S3–40 and S4–20 with overlap lengths of 20 and 40 mm were lower than that of specimen S2–300 reinforced by rebar without overlapping, mortar specimens reinforced with overlap lengths of 20 and 40 mm showed favorable flexural tensile strengths.

**Figure 20:**
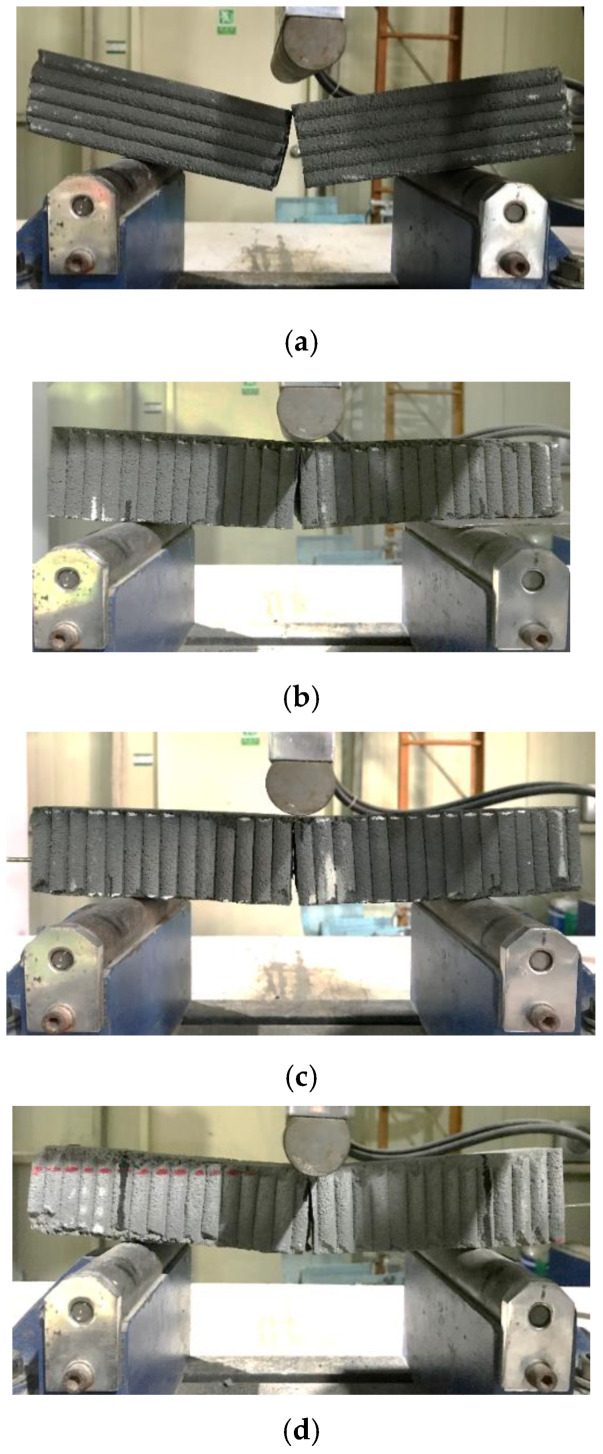
Flexural tensile failure patterns of mortar samples under loading directions I and III. (**a**) Specimen S1 (Loading direction I); (**b**) Specimen S2–300 (Loading direction III); (**c**) Specimen S3–40 (Loading direction III); (**d**) Specimen S4–20 (Loading direction III).

The flexural failure patterns of water-cured specimens under different loading directions are shown in Figure 20. The failures of mortar specimens without interlayer reinforcements occurred suddenly at the loading point, while the failures of mortar specimens with interlayer reinforcements were initiated by cracking at an interlayer near the loading point, which was followed by gradual widening of the crack. It was observed that the interlayer reinforcement was pulled out gradually at the failure interlayer, which meant that the interlayer reinforcements played a bridging role across the crack.

The flexural tensile strengths of air-cured samples under different loading directions are also shown in Figure 19. Without interlayer reinforcement, the flexural tensile strengths under loading direction III were considered the weakest. However, the flexural tensile strength of mortar specimen S2–300 under loading direction III was not smaller than that of mortar specimen S1 under loading directions I and II. The test results showed that reinforcements penetrating through 30 printed layers without overlapping in specimen S2–300 remarkably improved the load capacity of the specimen. Specifically, the flexural tensile strength of the printed specimen with longitudinal reinforcement without overlapping (S2–300) was 24.3% and 39.4% greater than those of the S3–40 and S4–20 specimens, respectively. This was due to the effect of the longitudinal reinforcement connecting all printed layers, thereby improving the interlayer bonding strength.

In addition, when the flexural strengths of specimens with different overlap lengths were tested under loading direction III, the flexural tensile strength of specimen S3–40 was greater than that of specimen S4–20. The load capacity of specimen S3–40 was 12.1% higher than that of specimen S4–20. The overlap length of 40 mm in specimen S3–40 was two times longer than the 20 mm length used in specimen S4–20. Therefore, the flexural tensile strength of specimen S3–40 was higher than that of specimen S4–20. The test results indicated that the flexural tensile strength of a printed specimen could be enhanced by interlayer reinforcement with a longer overlap length in the range analyzed in this study.

Regarding the failures of specimens cured under air, failure occurred as in the case of water curing, with a sudden crack at the loading point in the mortar specimen without reinforcement (S1). However, the failure of mortar specimens with reinforcement occurred by gradual widening of cracks at an interlayer close to the midspan of the prismatic specimen.

When the flexural tensile strengths under loading directions I and II were compared between specimens cured under water and air, the flexural tensile strengths of air-cured specimens were less than half of those of water-cured specimens. Under loading directions I and II, flexural tensile stresses did not act perpendicular to the interlayer interfaces of the mortar specimens. Therefore, the flexural tensile strength was governed by mechanical properties in the tensile zone of the mortar specimen and not by the strength of bonding at the interlayers. Curing conditions influenced the drying shrinkage of mortar. The drying shrinkage of mortar under air-curing conditions was greater than that under water-curing conditions. Accordingly, drying shrinkage in the tensile zone of the prismatic specimen decreased the flexural tensile strength of the printed mortar.

Additionally, under loading direction III, the flexural tensile strengths of specimens cured in air were significantly less than those of specimens cured under water. The test results showed that the load capacity of water-cured specimens was 47.6% greater than that of air-cured specimens, implying that the different curing conditions affected the flexural tensile strength. Curing conditions might affect the hydration process and pore formation at the interlayers in 3D-printed mortar.

Lee et al. [42] performed a study to determine the relationship between air pores and the tensile bonding strength of 3D-printed specimens. Using X-ray computed tomography, they found that the distributions of air pores at the interlayer contributed directly to the formation of cracks in 3D-printed specimens. Moreover, the physical and mechanical properties of 3D-printed mortar reinforced by E6 glass fibers were examined in the study by Shakor et al. [13]. Using a laser scanning microscope and ImageJ software to distinguish the embedded fibers, voids, and joint gaps on the cut surface of the sample by color, the joint gaps between two printed layers and voids were also analyzed in their study. Voids were distributed randomly in the printed layers and at the joint gap. They assumed that voids in printed mortar might be caused by the addition of admixture or the pumping process used to deliver the mortar during printing.

Therefore, air pores at the interlayers of 3D-printed mortar are more vulnerable under loading, and thus, the failure of printed mortar specimens under loading direction III could occur at a lower load than under the other loading directions considered in this study.

## 6. Conclusions

The interlayer bonding strengths of 3D-printed mortar with different curing conditions and reinforcement methods were investigated in this study. Based on extensive test results, the conclusions of this study can be drawn as follows:

- The compressive strength of the 3D-printed specimen produced with water-curing conditions under loading direction II (III) was 32.9% lower than that under loading direction I. The failure patterns of the specimens showed that debonding of the interlayers due to lateral deformation eventually caused the failures of the printed specimens under loading direction II.
- The splitting tensile failure of the 3D-printed mortar specimens under loading direction III occurred along the interlayer face, while the splitting tensile failure of the specimens under loading direction II occurred along the face perpendicular to the interlayer. Moreover, for specimens produced under water-curing conditions, the splitting tensile strength under loading direction III was 55% lower than that under loading direction I. Therefore, the splitting tensile strength was highly dependent on the loading direction.
- The splitting tensile strength resulting from water curing was greater than that resulting from air curing. Compared to those produced under air-curing conditions, the splitting tensile strengths of printed specimens produced under water-curing conditions increased by 28.9–72.7%. This indicated that water curing of the specimens promoted hydration of the mortar and thus improved the splitting tensile strengths of the specimens.
- The test results showed that the presence of reinforcements and the use of different overlap lengths affected the flexural tensile strength. The flexural tensile strength of the printed specimens increased by 12.5–39.4% when interlayer reinforcement was added. In addition, the flexural tensile capacity of the printed specimen with an overlap length of 40 mm was 12.1% greater than that of the printed specimen with an overlap length of 20 mm.
- The failure of 3D-printed specimens depended on the presence of interlayer reinforcement. The test results implied that interlayer reinforcements played a bridging role across cracks, and accordingly, the interlayer reinforcements were gradually pulled out at the failure interlayer.
- The flexural tensile strengths of the 3D-printed specimens produced under air curing conditions were 27–57% lower than those of specimens produced under water-curing conditions. This implied that possible air pores and joint gaps between printed layers would decrease the resistance to loading. Extensive analysis of the pore structures and joint gaps between 3D-printed layers was not performed in this study. However, the characteristics of microstructures in interlayers will be investigated in future studies.

## Figures and Tables

**Table 1:** Physical properties of the aggregates used.

W/B	Unit Weight (kg/m^3^)							Cementitious Paste Volume (%)
	Water	OPC	SF	FA	Sand	HWRA	Viscosity Agent	
0.25	222	610	87	175	1,206	17.4	0.87	52.0

Note: OPC: ordinary Portland cement, SF: silica fume, FA: fly ash, and HWRA: high-performance water-reducing agent.

**Table 2:** Strength properties of the 3D-printed mortar.

Strength	Fabrication Method	Loading Direction	Interlayer Reinforcement (mm)	Curing Conditions			
				Water Curing		Air Curing	
				Mean	S.D.	Mean	S.D.
				(MPa)	(MPa)	(MPa)	(MPa)
Compressive strength (*f_c_*)	Monolithic	-	-	75.3	4.3	45.5	2.3
	Printed	I	-	49.6	11.4	25.7	8.5
		II	-	33.3	5.3	25.3	1.2
		III	-	(33.3) *	(5.3) *	(25.3) *	(1.2) *
Splitting tensile strength (*f_t_*)	Printed	I	-	5.8	0.8	4.5	0.3
		II	-	3.8	0.4	2.2	0.4
		III	-	2.6	0.1	1.6	0.2
Flexural tensile strength (*f_r_*)	Printed	I	-	10.0	0.6	4.3	1.5
		II	-	10.3	0.7	4.5	1.1
		III	300 (no splice)	6.3	1.1	4.6	1.2
		III	40 (splice)	5.6	1.0	3.7	0.9
		III	20 (splice)	5.1	1.5	3.3	0.9

Note: S.D.: standard deviation, *: In the compressive strength test, loading direction II was the same as loading direction III. Therefore, the test results under loading direction II are also shown for loading direction III.

## Data Availability

The data used to support the findings in this study are available from the corresponding author upon request.
